# Advancing sexual health and well-being for all

**DOI:** 10.2471/BLT.24.292859

**Published:** 2024-12-01

**Authors:** Sagri Singh, Sahil Tandon, Uwemedimo Uko Esiet, Anna E Kågesten, Sofia Gruskin, SM Shaikat, Luchuo Engelbert Bain, Faysal El Kak, Carmen H Logie, Rajalakshmi RamPrakash, Mariangela Freitas da Silveira, Rayka Kumru, Zahra Stardust, Tarek Turk, Eusebio Rubio-Aurioles, E Tyler Crone, Thierry Troussier

**Affiliations:** a 27-A New Cantt Road, Hathibarkala, Dehradun 248001, Uttarakhand, India.; b Singapore, Singapore.; c The African Federation for Sexual Health and Rights, Lagos, Nigeria.; d Department of Global Public Health, Karolinska Institutet, Stockholm, Sweden.; e Institute on Inequalities in Global Health, University of Southern California, Los Angeles, United States of America (USA).; f SERAC-Bangladesh, Dhaka, Bangladesh.; g African Population and Health Research Center, Nairobi, Kenya.; h Department of Obstetrics and Gynecology, American University of Beirut Medical Center, Beirut, Lebanon.; i Factor-Inwentash Faculty of Social Work, University of Toronto, Toronto, Canada.; j Chennai, India.; k Faculdade de Medicina, Universidade Federal de Pelotas, Pelotas, Brazil.; l Istanbul, Türkiye.; m Queensland University of Technology, Brisbane, Australia.; n Department of Psychiatry, University of Alberta, Edmonton, Canada.; o Mexican Association for Sexual Health, Mexico City, Mexico.; p Seattle, USA.; q Sexual Health and Human Rights, United Nations Educational, Scientific and Cultural Organization, Paris, France.

In the past 50 years, sexual and reproductive health and rights have been increasingly recognized as critical components of overall health and well-being.^1^^,^^2^ The 1975 World Health Organization (WHO) working definition of sexual health highlighted the need for actions to improve women’s and girls’ sexual health to realize gender equality. This definition of sexual health emphasizes not only physical well-being but also a positive, respectful approach to sexuality, ensuring safe, pleasurable experiences free from coercion, discrimination and violence, with sexual rights respected, protected and fulfilled for all persons.^3^

Despite the universality of sex and sexuality to human experience,^4^ much work remains to normalize sexual health and well-being and prioritize it in public health and development agendas. Normalization requires challenging inequitable social norms regarding sexuality, particularly reducing stigma and advancing sexual rights and justice, especially for women, girls, and persons of diverse sexual orientation, gender identity and expressions, and sex characteristics. 

The low prioritization of sexual health and well-being as a fundamental human rights and justice issue in development and health agendas results from years of neglect in public health discourse due to pervasive stigma, controversy and misunderstanding.^5^^,^^6^ A disease-focused, harm-prevention and treatment approach to sexual health and well-being (such as prevention of sexually transmitted infections) disregards the multidimensional realities of sexuality across the life course.^5^ This approach has also contributed to considerable gaps in research, policies, programmes and funding and a need to explain how sexual health differs from reproductive health.^7^^,^^8^

An anti-gender movement and political conservatism^9^^,^^10^ are dividing those working to advance sexual and reproductive health and rights, gender equality, equity, health and human rights.^11^ Fulfilling sexual health and well-being as a fundamental right necessitates addressing social and structural determinants to prevent harm and foster a positive approach that ensures physical and mental wellness, sexual autonomy, inclusivity, pleasure and rights. 

WHO has established a Sexual Health and Well-being Advisory Group, aimed at broadening the approach to a sexual health and well-being agenda. As members, our first step is to gather a knowledge base anchored in diverse experiences that link sexual well-being with human development and rights. This step involves a learning agenda, identifying metrics to distinguish sexual health and well-being, and increasing collective engagement and action to promote normalization of sexual health. As a global community, we must address the full consequences of failing to realize sexual health and well-being and its socioeconomic implications, to make a case for investment, advocacy and policy prioritization. 

We invite the global communities of practice, civil society partners, local and regional networks and policy advocacy champions to shape and support these efforts. Our collective work can lead to increased visibility and engagement on these issues, creating messages and approaches that are adaptable and inclusive of diverse contexts and that can build positive, rights-based narratives and policies, stimulating support for a broader movement that recognizes sexual health and well-being in health and development agendas.
